# Multicomponent analysis of T_1_ relaxation in bovine articular cartilage at low magnetic fields

**DOI:** 10.1002/mrm.27624

**Published:** 2018-12-10

**Authors:** Oleg V. Petrov, Siegfried Stapf

**Affiliations:** ^1^ Ilmenau University of Technology Ilmenau Germany

**Keywords:** articular cartilage, enzymatic digestion, inverse Laplace transform, logarithmic moment analysis multiexponential relaxation, T_1_ relaxation

## Abstract

**Purpose:**

The multi‐exponential character of T_1_ relaxation in bovine articular cartilage was investigated at low magnetic fields below 0.5 T. The ultimate aim was to identify a parameter based on the T_1_ relaxation time distribution as a biomarker to biochemical features of osteoarthritis.

**Methods:**

Osteoarthritis conditions were simulated by enzymatic digestion of cartilage with trypsin. Fast‐field cycling NMR relaxometry was carried out in the magnetic field range B_0_ = 70 μT to 600 mT. The data were analyzed in terms of T_1_ distributions on a log‐time scale using inverse Laplace transform, whereas integral properties such as mean T_1_s and distribution widths were obtained without data inversion from logarithmic moment analysis and a stretched‐exponential fit to the data. Attempts were also made to differentiate between water dynamic components through multi‐Lorentzian decomposition of average relaxation‐rate dispersions.

**Results:**

T_1_ distribution in bovine articular cartilage was found to be bimodal, with the dominating, long component shifting toward larger values following trypsin digestion. The effect is more prominent toward lower magnetic field strength. This shift leads to an overall increase of the distribution width and an equivalently more pronounced deviation from exponential behavior.

**Conclusion:**

The logarithmic width of T_1_ distribution functions at fields of 0.5 T and below, and the stretched‐exponential decay fit exponent *β*, show a significant trend after trypsin digestion of cartilage. These 2 parameters are suggested as possible biomarkers for osteoarthritis in humans and can be acquired entirely in vivo, with increasing significance for lower magnetic field strengths.

## 1 INTRODUCTION

Articular cartilage (AC) is a thin layer of connective tissue that covers and protects the articular surfaces of bones. One form of degeneration of articular cartilage is called osteoarthritis (OA), which is one of the major causes of mobility disability among the elderly. The MRI techniques developed for early diagnosis of OA exploit spatially resolved measurements of T_1_, T_2_, and T_1ρ_ as potential biomarkers, thanks to their correlation with the composition and structure of AC.^1^ In addition to the MRI mapping of T_1_ and T_2_, fast field‐cycling (FFC) NMR relaxometry has recently been used in the attempt to correlate the biological state of AC cartilage with T_1_ dispersion.^2^, ^3^, ^4^ The FFC experiments were carried out in the magnetic fields B_0_ = 0.2 mT to 470 mT, which span quadrupolar relaxation enhancement (QRE) peaks. A statistically significant difference was found between osteoarthritic and healthy cartilage for both the dispersion amplitude and the area of the QRE peaks,^2^, ^4^ as well as for the maximum values of T_1_ and T_2_ when the samples were subject to external load.^4^ The power‐law exponent α in $T_{1}∼B_{0}^{\alpha }$ at B_0_ < 25 mT was found to correlate weakly with Mankin grade but significantly with other biomarkers.^5^ A number of parameters obtained at low magnetic field strengths were found to correlate with disease status only under external load.^4^

In this work, we aim to analyze FFC relaxation data in terms of a T_1_ distribution, *g*(*T*_1_), underlying a total magnetization relaxation function *M_tot_*(*t*). Our particular goal was to investigate the field dependence of *g*(*T*_1_) and see whether it is affected by the enzymatic treatment of cartilage that mimics the early stage of OA (normal versus OA‐relevant degraded tissue) and thus has the potential of being a biomarker for OA.

**Figure 1:**
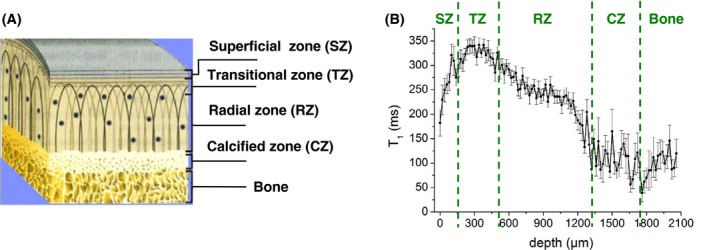
A, Four zones of articular cartilage based on orientation of collagen fibers and concentration of water and proteoglycans.^14^ B, Spatially resolved measurements of T_1_ relaxation times in the stray field of a single‐sided NMR scanner NMR MOUSE, at a Larmor frequency of 11.7 MHz (reprinted from Ref 5). In the original paper,^5^ single‐exponential fits were carried out per slice

Nonexponential T_1_ relaxation in AC can be anticipated from its zonal structure. Indeed, spatially resolved measurements of T_1_ on bovine hip’s cartilage samples at B_0_ = 0.27 T^5^ have shown that T_1_ ranges between 30 ms in the calcified zone and 350 ms in the transitional zone, which is over 1 order of magnitude (Figure 1). Therefore, one might expect a multiple T_1_ relaxation for *M_tot_*(*t*) over the entire range of B_0_ covered by a typical FFC instrument (B_0_ ≤ 0.6 T), or at least below 0.27 T where this observation was made.

In practice, nonexponential NMR relaxation is analyzed using inverse Laplace transform (ILT). A number of ILT algorithms are available^6^, ^7^, ^8^ that convert *M*(*t*) into a histogram of T_1_, which then can be submitted to further statistical analysis or used as a relaxation metric in itself. In this work, the ILT is used primarily for selective T_1_‐component monitoring, whereas statistical (integral) properties of distributions such as the geometric mean T_1_ and the geometric standard deviation *σ_g_* are obtained without data inversion from a logarithmic moment analysis (LMA).^9^ An alternative approach to integral measures of *g*(*T*_1_) is available from a stretched‐exponential fit to *M*(*t*), which gives the arithmetic mean T_1_ and a stretching exponent as the measure of the degree of nonexponentiality. All 3 methods are complementary to each other and used here in comparison, with the aim of testing their robustness toward routine measurements.

A shape analysis of T_1_‐relaxation dispersion can assist in probing the multicomponent relaxation in the frequency domain. With a good accuracy, a correlation time distribution, *g*(*τ_c_*), can be obtained through a multi‐Lorentzian decomposition of a given relaxation rate profile. This sort of analysis assumes that the relaxation rate at every B_0_ is a sum of rates of elementary relaxation processes driven by fluctuations with exponential correlation functions. We will refer to such processes as Bloembergen‐Purcell‐Pound–type relaxation. Such a decomposition is generally possible mathematically, and is expected to be robust, regardless of the underlying correlation functions and detailed molecular dynamics models. The formalism of the multi‐Lorentzian decomposition is introduced here instead of the power‐law approximation to the T_1_ dispersion^4^ in the attempt to draw a correlation between *g*(*τ_c_*) and the changes caused by enzymatic treatment of cartilage.

## 2 METHODS

### 2.1 Fast field cycling NMR relaxometry

**Figure 2:**
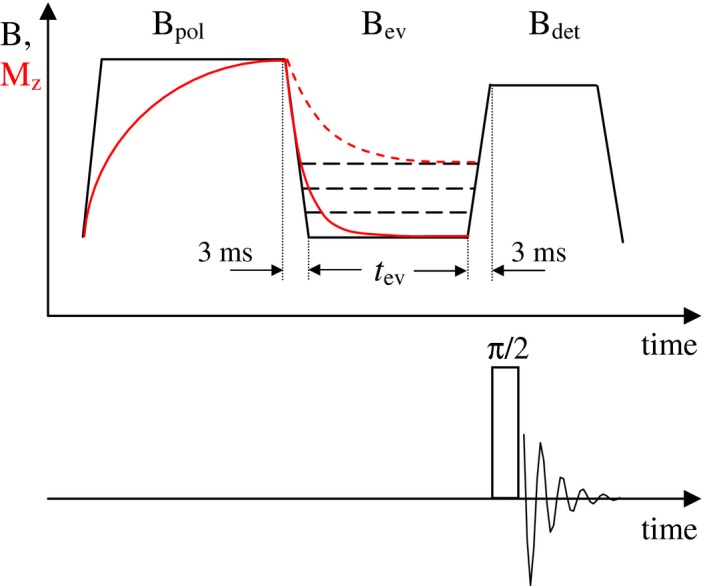
Protocol of a fast field‐cycling (FFC) relaxometry experiment with a prepolarization pulse. The field switching time was set to 3 ms. Black lines indicate magnetic field variations and the red line corresponds to the evolution of the magnetization

T_1_‐relaxation decays were recorded on a commercial field‐cycling relaxometer Spinmaster FFC2000 (Stelar s.r.l., Mede, Italy) in the magnetic field range B_0_ = 70 μT to 600 mT corresponding to the ^1^H Larmor frequency $\nu_{0}$ = 3 kHz to 25 MHz. The minimum value of 3 kHz is beyond the lower limit of 10 kHz recommended by the relaxometer manufacturer, given background magnetic fields. However, because dispersion profiles does not exhibit a tendency toward a plateau below 10 kHz, as can be expected if the background fields predominate, it appears appropriate to keep the corresponding data, provided greater care is taken. The magnetic field varied from the highest value downward in logarithmically spaced steps. The number of steps was 20 when scanning over the entire field range and 50 for a separate scan over the QRE region ($\nu_{0}$ = 0.4 MHz‐4 MHz). A pulse sequence with a prepolarization pulse was used at $\nu_{0}$ < 5 MHz, which produced *M*(*t*) in a form of decay (Figure 2), and without prepolarization above 5 MHz in which case *M*(*t*) was a recovery function. The relaxation delay *t*_ev_ varied from 0.01 to 5‐10 × T_1_, in 24 logarithmically equidistant steps. With 4 averaging scans per delay, a 20‐point profile was acquired under 2 hours. The FID acquisition field was fixed at 0.4 T ($\nu_{0}$ = 16.7 MHz), the 90° RF pulse length being 7.5 μs. The field‐switching time was set to 3 ms (Figure 2) and the slew rate to 9 MHz/ms. The temperature of the samples was 10°C.

### 2.2 Multi‐exponential data analysis

The inverse Laplace transformation of *M*(*t*) was performed by the program UPEN.^7^ The algorithm of UPEN features an uniform smoothing penalty over the sharp and broad components of a distribution. The calculated distributions are supplied in the form of histograms of T_1_ with logarithmically sized bins.

The geometric mean T_1_, <*T*_1_>*_g_*, and the geometric SD of T_1_, *σ_g_*, were calculated without data inversion using an LMA. The LMA algorithm consists, in essence, of a numerical integration of *M*(*t*) that is preliminary normalized to [1, 0], followed by a few elementary operations.^9^ By definition,

(1) $$\sigma_{g}=\exp\left(\sqrt{\sigma_{\ln T_{1}}^{2}}\right),$$

where $\sigma_{\ln T_{1}}^{2}$ is the variance of T_1_ on the logarithmic time scale. The lower limit of *σ_g_* is 1, which corresponds to a single exponential T_1_ relaxation. The logarithm of *σ_g_* is proportional to the width of a T_1_ distribution when the latter is plotted against the logarithm of T_1_. This makes *σ_g_* a convenient parameter to be used alongside the ILT histograms with a logarithmical abscissa. For a unimodal, symmetrical histogram, $\sigma_{g}-1$ is approximately equal to the decadic logarithm of the ratio of the largest to the smallest T_1_ at half maximum; as such, $\sigma_{g}-1$ measures the T_1_ distribution in terms of decades (or orders of magnitude).

The stretched‐exponential fit is a simple phenomenological model

(2) $$M\left(t\right)=A\exp\left[-{\left(t/\tau_{K}\right)}^{\beta }\right]+M_{\infty },$$

in which the stretching exponent *β * [0, 1] measures the deviation from a single exponential (inversely correlated with *σ_g_*). The arithmetic mean T_1_, <*T*_1_>*_a_*, can be conveniently calculated from *β *and *τ_K_* as

(3) $$<T_{1}{>}_{a}=\left(\tau_{K}/\beta \right)\;\Gamma \left(1/\beta \right),$$

where Γ denotes the gamma function. This stretched‐exponential fit is robust and less demanding for the completeness of the data compared with the LMA; however, it does not suit the case of distributions with well‐resolved and sharp T_1_ components. Because Equation 2 is a phenomenological model, the parameter *β* is correlated with *σ_g_* only qualitatively, as well as with other measures that depend on the second moment of a distribution.

### 2.3 Multi‐Lorentzian data analysis

This analysis applies to variable‐field measurements of the arithmetic mean relaxation rate $<R_{1}{>}_{a}$. By definition, $<R_{1}{>}_{a}=1/<T_{1}{>}_{h}$ where $<T_{1}{>}_{h}$ is the harmonic mean T_1_. In principle, $<T_{1}{>}_{h}$ can be calculated from ILT histograms of T_1_. However, this scheme was found to introduce a large scattering in values and therefore is considered impractical. Instead, we recommend using the approximation $<T_{1}{>}_{h}\;\approx \;\;<T_{1}{>}_{g}^{2}/<T_{1}{>}_{a}$,^10^ where the geometric $<T_{1}{>}_{g}$ and the arithmetic $<T_{1}{>}_{g}$ means are taken from the LMA and the stretched‐exponential fit, respectively. Note that the multi‐Lorentzian analysis of dispersion profiles is also applicable when relaxation functions are approximated by a single exponential. Although being of limited utility in case of a T_1_ distribution, the single‐exponential fit to *M*(*t*) gives the least scattered dispersion profiles among the present parameterization methods (see subsequently).

Henceforth the brackets and subscript “*a*” in $<R_{1}{>}_{a}$ is omitted for simplicity. The model for $R_{1}\left(\nu_{0}\right)$ is a weighted sum of *N* contributions of Bloembergen‐Purcell‐Pound type, representing uncorrelated random processes with correlation times $\tau_{c,i}$:

(4) $$R_{1}\left(\nu_{0}\right)=\sum_{i=1}^{n}p_{i}\left(\frac{0.2\tau_{c,i}}{1+4\pi^{2}\nu_{0}^{2}\tau_{c,i}^{2}}+\frac{0.8\tau_{c,i}}{1+16\pi^{2}\nu_{0}^{2}\tau_{c,i}^{2}}\right)\;\approx \;\sum_{i=1}^{n}p_{i}\frac{\tau_{c,i}}{1+12\pi^{2}\nu_{0}^{2}\tau_{c,i}^{2}}$$

which is, with a high accuracy, approximated by a sum of single Lorentzian dispersions with an effective correlation time ${\tilde{\tau }}_{c}=\sqrt{3}\tau_{c}$.^11^ The approximation in Equation 4 enables a 2‐step inversion of $R_{1}\left(\nu_{0}\right)$. First, it is Fourier transformed to represent data as a sum of exponentials in the time domain, and then it is submitted to a multi‐exponential decomposition by means of ILT. Because $R_{1}\left(\nu_{0}\right)$ is measured at logarithmically equidistant frequencies, a special Fourier‐transform algorithm called FFTLog is used.^12^

### 2.4 Sample preparation

Samples of bovine articular cartilage were taken from calf knee joints by slicing them to a depth of several centimeters along the bone direction with the aid of a commercially available bow saw. The thickness of the slices was approximately 4 mm. A cartilage layer was separated from the subchondral bone by using a manual cutting machine. The cut chips of cartilage were 5 mm to 10 mm long, depending on the radius of curvature of the joint’s surface. To keep the whole of the calcified zone, which is only a few hundred micrometers thick and has a wavy surface, we had to cover a certain part of the adjacent bone when cutting. Each chip was then visually examined, and those containing a considerable amount of the subchondral bone were discarded. Thus, we do not expect bone marrow, if any, to contribute significantly to the NMR signal. A dozen or so of cartilage chips collected from 1 spot of the knee joint (either at a lateral condyle or a tibia) constituted 1 NMR sample.

If no enzymatic treatment was applied, the cartilage chips were kept in pure physiological saline solution (phosphate buffered saline) for 24 h under constant agitation at room temperature. The chips were removed from the phosphate buffered saline 2 hours before the NMR measurement, put in a Petri dish, and kept unsealed in the refrigerator to remove excess surface moisture. Then they were packed into a 10‐mm NMR tube and covered with Fluorinert FC‐70 fluid (a fully fluorinated aliphatic compound) to preserve the state of the cartilage. Once the NMR relaxometry experiments were complete, the cartilage chips were removed from the NMR tube, washed in pure phosphate buffered saline, and then placed in a phosphate buffered saline containing trypsin (Sigma‐Aldrich, T1426, 0.5 mg/mL concentration), where they were kept at 34°C under constant agitation for 24 hours. Afterward, the cartilage underwent this wash‐and‐dry procedure and was placed back to the NMR tube and covered with Fluorinert for the second run of the FFC measurements.

The cartilage chips were packed into an NMR tube closely and stacked there nearly parallel to each other. This might give a preference to a particular orientation of collagen fibers with respect to B_0_. Previous measurements,^13^ however, have not revealed a significant angular dependence of T_1_ in bovine cartilage; thus, the orientation of cartilage specimens in an NMR tube will not introduce any ambiguities in the T_1_ distribution measurements.

Overall, 3 calf knees were used that had been supplied by a local butcher on demand. The number of NMR samples made from a given calf knee varied from 2 to 4. No systematic documentation between origin and position of these samples was carried out, so these will be denoted symbolically as “sample A, B, C, …” in the order of their mentioning in the text.

## 3 RESULTS

### 3.1 Individual T_1_ component monitoring

**Figure 3:**
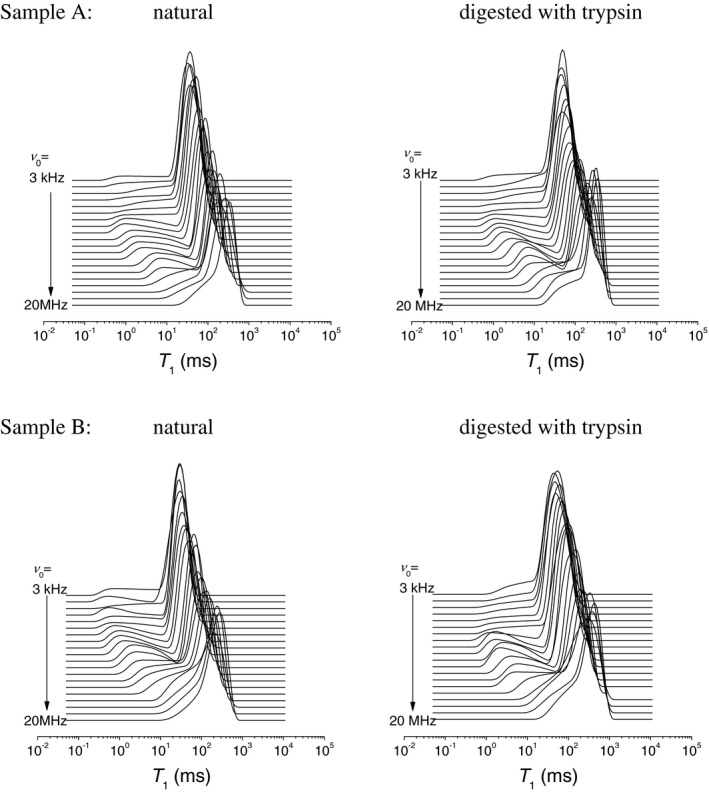
Representative T_1_ distributions in bovine articular cartilage before and after trypsin digestion, at different magnetic field strengths, indicated by their Larmor frequencies *ν*_0_

Figure 3 shows representative T_1_ distributions in bovine articular cartilage at a variable $\nu_{0}$. They exhibit two partially overlapping components with significantly dissimilar field dependences (dispersions). The components become fully resolved at $\nu_{0}$ = approximately 2 MHz, at which point they appear to constitute 20% and 80% of the population, respectively. As the field lowered into a sub‐megahertz region, the short‐time edge of the minor peak eventually reaches the instrumental limit of measurable T_1_ values (approximately 1 ms), which decreases its apparent amplitude down to 10%.

**Figure 4:**
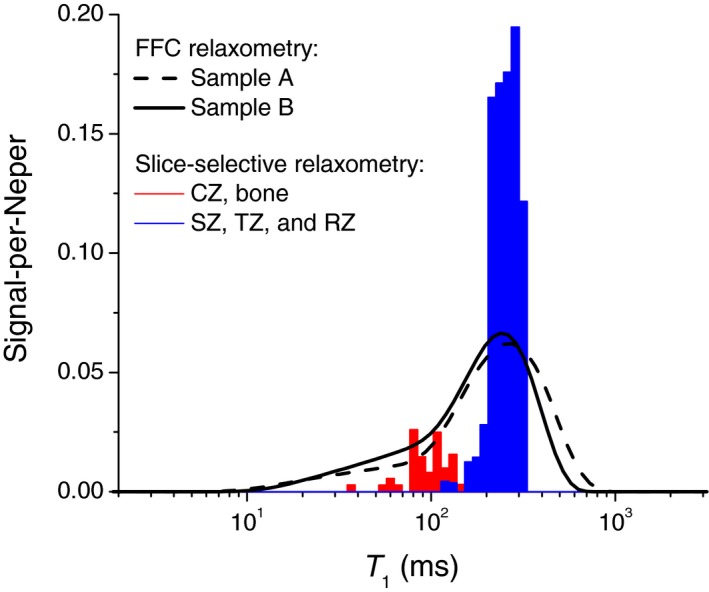
Comparison of the T_1_ distribution measurements from FFC relaxometry (the solid and dashed lines) and slice‐selective relaxation data presented in Figure 1. The experiments were carried out at *ν*_0_ = 12.5 MHz and 11.7 MHz, respectively. To plot a histogram based on the slice‐selective data, the counts of T_1_ were weighted using a corresponding NMR signal intensity profile.^14^ The contribution from the calcified zone and the underlying bone is highlighted in red

To relate the observed T_1_ components to the constituents of cartilage, we recapitulated the results of the spatially resolved measurements of T_1_ by Rössler et al^5^ carried out on a bulk sample of bovine articular cartilage. Thus, we plotted a histogram of the T_1_ values from that study across the cartilages (Figure 1B), weighting their counts with the respective signal intensities^14^ and then overlaid it with our measurements (Figure 4). Remarkably, the T_1_ distributions from the two sources exhibit the same peak’s positions and comparable areas on the left sides from the peak. This supports the idea that the T_1_ variance across zones is a main factor of the T_1_ distribution observed in our FFC experiments. Because the data analysis used single exponential functions exclusively,^5^ and a certain structural and/or dynamic heterogeneity is expected even within the thin slice, the actual distribution of T_1_ values must indeed be wider. The T_1_ components on the left‐hand side originate mostly from the calcified zone and underlying bone (see red‐colored bars in Figure 4), which accounts for about 11% of the total histogram area. This allows us to ascribe the minor T_1_ component of the ILT histograms in Figure 3 primarily to the calcified zone. The comparatively short T_1_ and a stronger T_1_ dispersion of this component indicate a lower molecular mobility of water in that part of cartilage.

It is apparent from the FFC data that T_1_ relaxation tends toward a single exponential as the field increases. To ascertain this tendency, we complemented the FFC data with conventional inversion‐recovery T_1_ relaxometry carried out at $\nu_{0}$ = 43.4 MHz (Spinsolve Benchtop NMR, Magritek Ltd, Wellington, New Zealand). The T_1_ distributions at this field were found to collapse to an irresolvable narrow peak situated within T_1_ = 475 ms to 545 ms, depending on the cartilage sample measured.

**Figure 5:**
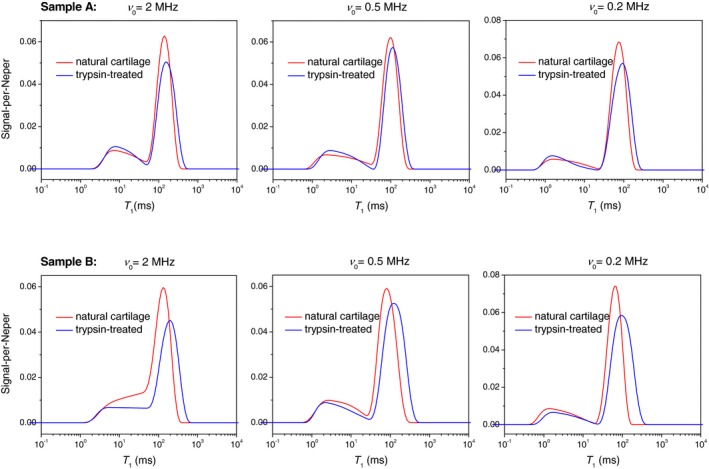
Representative inverse Laplace transform (ILT) histograms of T_1_ illustrate the effect of trypsin digestion on T_1_ populations in articular cartilage. It is apparent that the effect consists primarily of a shift of the long T_1_ peak (which is considered a cumulative signal from superficial, transitional, and radial zones) toward longer times

Trypsin digestion of the cartilage does not appear to have an appreciable effect on the minor T_1_ component of the distribution (assigned primarily to a signal from the calcified zone). However, this leads to a shift of the major T_1_ component toward longer T_1_ values; thus, it increases the contrast between the two T_1_ populations (Figure 5). The effect of trypsin on the average values of T_1_, $<T_{1}{>}_{g}$ and $<T_{1}{>}_{a}$, may vary among cartilage samples,^15^ but overall the T_1_ relaxation becomes slower after trypsin digestion at all $\nu_{0}$ in the given FFC frequency range. This agrees with previous FFC investigations of enzymatically degraded cartilage,^3^ in which this fact was established from a single‐exponential fit to *M*(*t*). A similar observation has been reported for the transverse relaxation time components.^16^

### 3.2 Integral parameters of T_1_ relaxation

**Figure 6:**
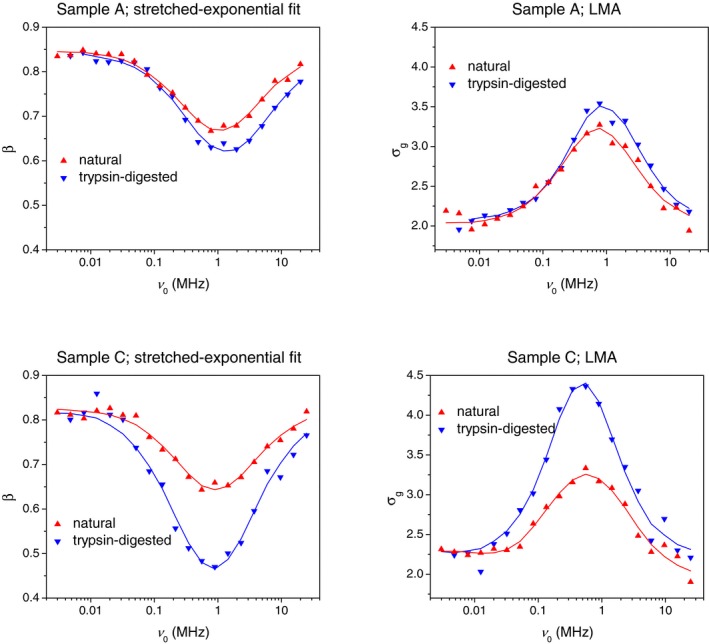
Two metrics of nonexponential T_1_ relaxation—the geometric SD *σ_g_* and the stretching exponent *β*—as a function of *ν*_0_. Lowering *ν*_0_ increases the degree of nonexponentiality, which is securely detected down to *ν*_0 _= 1 MHz. Below 1 MHz, the shortest T_1_ components become too short for a parity detection with longer T_1_ components, hence the apparent extremum. Digestion of cartilage with trypsin increases the degree of nonexponentiality. The solid lines are guides to the eye. Abbreviation: LMA, logarithmic moment analysis

Figure 6 shows the T_1_ distribution width parameters *σ_g_* and *β* obtained through the LMA and the stretched‐exponential fit to *M*(*t*), respectively. Both *σ_g_* and *β* have a prominent extremum at about $\nu_{0}$ = 1 MHz in agreement with preliminary findings.^17^ The plots of the complete distribution functions in Figure 3 suggest that this extremum can be, at least partially, caused by the minor T_1_ component losing its amplitude when approaching the low‐field region. This in turn is a result of an irrevocable relaxation loss of the NMR signal during field switching intervals (Figure 2). Supplementary FFC NMR relaxation experiments carried out on synthetic samples have shown that in the case of two well‐separated T_1_ components, the ratio of the short‐T_1_ to the long‐T_1_ component begins to deviate noticeably from the true value when the shorter T_1_ is approximately one order of magnitude longer than the switching time (3 ms in this study).

Despite these instrumental restrictions on quantitation of short T_1_ components, the effect of trypsin treatment is securely detected through the integral distribution parameters *σ_g_* and *β*, indicating the broadening of T_1_ distributions in the enzymatically degraded cartilage.

### 3.3 Multi‐Lorentzian analysis of dispersion profiles

In addition to the apparent narrowing of the T_1_ distribution, the uneven relaxation losses of T_1_ components on field switching also bias the means, $<T_{1}{>}_{a}$ and $<T_{1}{>}_{g}$, in favor of longer T_1_s. Using them as such for calculating the arithmetic mean R_1_ leads to distorted dispersion profiles that can no longer be modeled as a sum of Lorentzian functions (see Equation 4). For this reason, instead of calculating the mean R_1_, we analyze the reciprocals of $<T_{1}{>}_{a}$ and $<T_{1}{>}_{g}$ as individual parameters. The reciprocal of a single‐exponential fit parameter ${\text{T}}_{1,mono}$ has also been considered. Strictly speaking, the multi‐Lorentzian decomposition of such profiles can no longer be interpreted in terms of superposing R_1_ dispersions. Rather, it is thought of merely as a parameterization technique that might be suitable to trace the changes caused by enzymatic treatment of cartilage.

**Figure 7:**
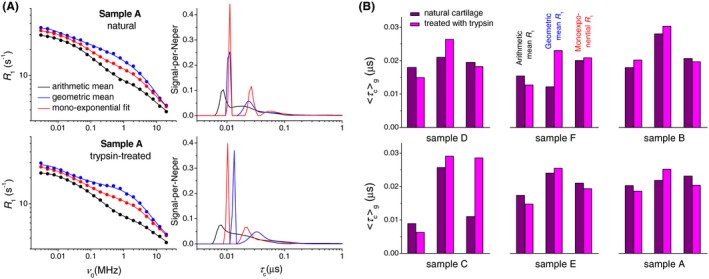
A, Dispersion profiles of three estimates of an average relaxation rate *R*_1_—the reciprocal of the arithmetic mean T_1_, of the geometric mean T_1_, and of the best‐fit single‐exponential decay rate. The solid lines represent the multi‐Lorentzian models (Equation 4), which are plotted using the correlation time distributions $g({\tilde{\tau }}_{c})$ shown on the right. B, Mean correlation times $<{\tilde{\tau }}_{c}{>}_{g}$ computed from the given $g({\tilde{\tau }}_{c})$ for each of the three R_1_ estimates. The means based on the $<T_{1}{>}_{g}^{}$ values (see the blue profiles) consistently increase after the trypsin digestion of cartilage

Figure 7A shows multi‐Lorentzian best‐fit curves and corresponding correlation time distributions $g({\tilde{\tau }}_{c})$ for sample A. The thus‐measured distributions cover more than 1 order of magnitude, from several to a hundred nanoseconds. The interpretation of the obtained $g({\tilde{\tau }}_{c})$ is out of scope of this work. We can only assume that a peak at ${\tilde{\tau }}_{c}\approx 10\;\text{ns}$, corresponding to a dispersion midpoint frequency $\nu_{0}$ 10 MHz, reflects internal motions in the aqueous gel (“bulk water”) or in the protein network,^11^ whereas the long‐${\tilde{\tau }}_{c}$ components account for relaxation of protein‐entrapped water (see subsequently).

To quantitate the effect of trypsin digestion on $g({\tilde{\tau }}_{c})$, we calculated the mean ${\tilde{\tau }}_{c}$ for all 3 cases of $<T_{1}{>}_{a}$, $<T_{1}{>}_{g}$, and ${\text{T}}_{1,mono}$ (Figure 7B). Only the $<{\tilde{\tau }}_{c}>$ based on the $<T_{1}{>}_{g}^{}$ measurements (the blue profiles in Figure 7A) demonstrated a consistent response (always increases) to trypsin digestion, whereas the two other estimates have no definitive trend.

### 3.4 T_1_ relaxation broadening at QRE peaks

**Figure 8:**
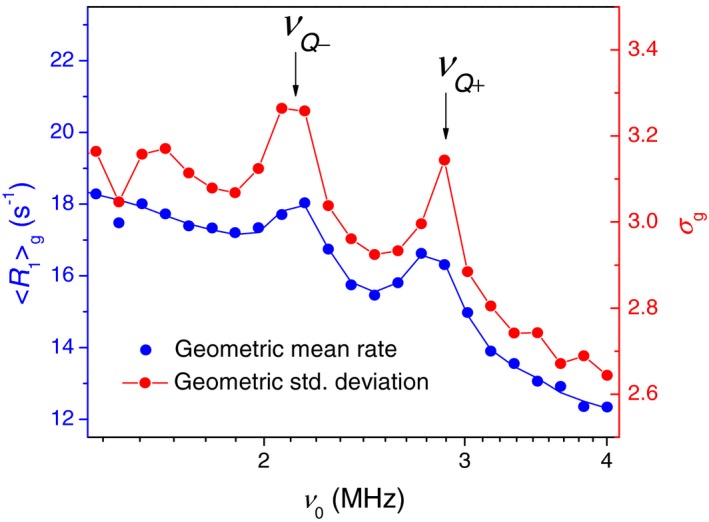
Geometric mean relaxation rate, $<R_{1}{>}_{g}$, and geometric SD, $\sigma_{g}$, measured with a higher resolution over a quadrupolar relaxation enhancement region (sample B). The arrows indicate 2 of 3 quadrupolar relaxation enhancement (QRE) peaks occurring at frequencies $\nu_{Q_{-}}$ and $\nu_{Q_{+}}$ of the static residual quadrupole coupling of amide ^14^N in immobilized proteins^18^

Figure 8 shows a $<T_{1}{>}_{g}^{-1}$ profile recorded with a higher resolution to cover two quadrupolar peaks on the high‐frequency side. Most interestingly, the peaks on the $<T_{1}{>}_{g}^{-1}$ profile are accompanied by peaks on a SD profile, $\sigma_{g}$, which is indicative of a higher degree of nonexponentiality of *M*(*t*) at the QRE frequencies.

The QRE is caused by a ^1^H–^14^N cross relaxation in the protein amide groups.^18^ It occurs under level‐matching conditions in the range of 0.4 MHz to 4 MHz, giving rise to three characteristic peaks on a ^1^H dispersion profile. Magnetization transfer from amide protons to the bulk water relies on diffusion and chemical proton exchange. If those processes are slow on the T_1_ time scale, one may indeed expect to observe an additional T_1_‐relaxation broadening at the QRE frequencies.

In general, the magnitude of the QRE peaks—of which often only the two high‐field peaks are sufficiently well pronounced to allow quantitative analysis—provide information about the number density and mobility of amide moieties, hence the concentration and mobility of proteins. It is a well‐known fact that proteoglycans are depleted during OA, and trypsin digestion is considered a suitable model for this aspect of OA degradation. This has been exploited to quantify the effect of trypsin digestion by measuring the QRE peak areas.^3^ The motive for presenting QRE data in this work is different: It is to show that the partition of water in AC into protein‐bound and bulk states is a sufficient cause of a T_1_ distribution on its own. We will use this argument in the Discussion to assess various sources of multiple T_1_ relaxation in cartilage.

## 4 DISCUSSION

These results show that the T_1_ distribution has the potential of being a biomarker for OA, provided that a suitable parameterization procedure is used. The result shown in Figure 6 is key: It demonstrates that trypsin digestion of cartilage has a pronounced effect on T_1_, which is securely detected over the entire $\nu_{0}$ range through the parameters *σ_g_* and *β*. On the ILT histograms (Figure 5), the trypsin digestion manifests in a shift of the longer and dominating T_1_ component toward longer T_1_ values. The maximum possible contrast between naturally treated and trypsin‐treated cartilage is achieved for the given FFC instrument at $\nu_{0}$ = approximately 1 MHz.

In the absence of a detailed model for T_1_ distribution that would account for both dynamical and compositional heterogeneity of AC, the discussion of these results is largely conjectural. At this point, one needs to discern and acknowledge all possible factors leading to the nonexponential T_1_ relaxation observed at low fields. The measured T_1_ relaxation pertains to water proton relaxation; therefore, both the factors of the T_1_ distribution and the changes observed in T_1_ dispersion need to be considered in the context of the particular conditions that water is in.

**Figure 9:**
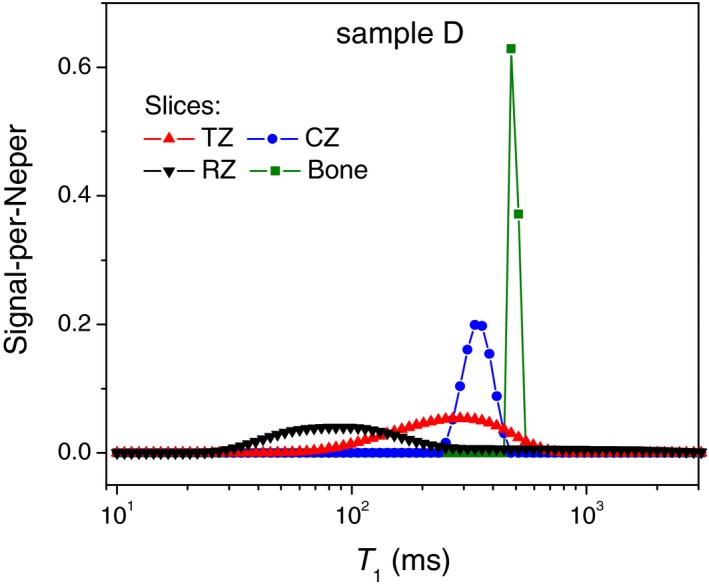
T_1_ distributions computed from slice‐selective T_1_ relaxometry with the NMR MOUSE (the measurements were performed by Dr. Andrea Creţu on a 1‐mm‐thick articular cartilage). Different colors denote morphological zones of articular cartilage being scanned over the course of the experiment (for zone acronyms, see Figure 1A)

**Figure 10:**
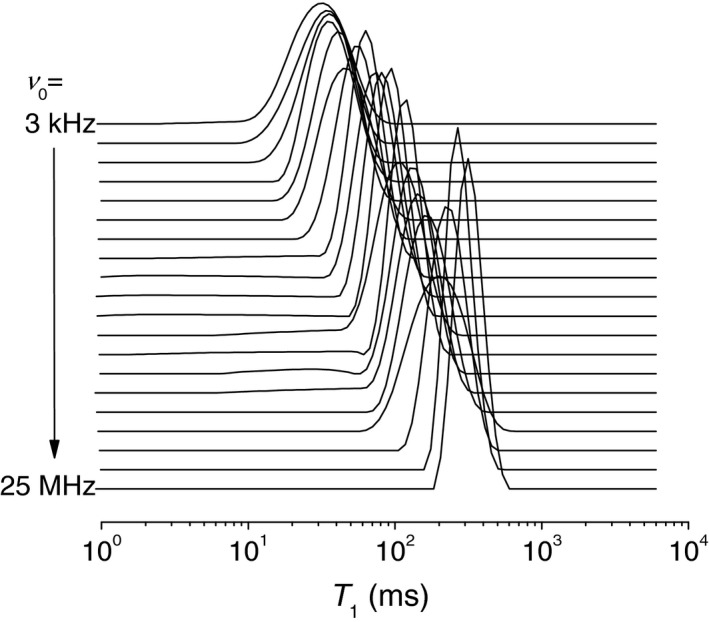
T_1_ distributions in bovine meniscus tissue as a function of *ν*_0_

From the slice‐selective low‐field relaxometry by Rössler et al,^5^ a single‐exponential‐fit parameter T_1_ varies throughout the depth of AC over one order of magnitude. Our reproduction of a log‐binned histogram of T_1_ (Figure 1B and Figure 4) showed that it had a similar shape to the ILT T_1_ distribution measured on the sum magnetization, even though it does not represent T_1_ values on the order of the field‐switching time with correct weighting.^5^ On this basis, we have to ascribe a major effect to the zonal heterogeneity of AC. However, one cannot exclude the T_1_ relaxation broadening due to water‐protein interactions on a *local *length scale, which refers to the magnetization transfer between water entrapped inside the protein matrix and bulk water.^19^ Thus, according to Ref 20, 30%‐35% of water in AC is entrapped in the intrafibrillar space within collagen and the remainder in the extrafibrillar space. These two domains may represent distinguishable relaxation sinks, provided an exchange between them is slower than individual relaxation. Indeed, the rise of $\sigma_{g}$ observed at the QRE frequencies (Figure 8) suggests that the relaxation enhancement due to water/protein contacts may not be completely averaged at $\nu_{0}$ of several megahertz and lower. In the attempt to single out this factor of T_1_ distribution, we analyzed the available data from slice‐selective T_1_ relaxometry with the slice width of 0.1 mm at $\nu_{0}$ = 11.7 MHz. The result turned out to be dependent on the position inside the tissue. Namely, we were able to detect a T_1_ distribution of finite width in the radial and the calcified zones, but not in the transitional zone (Figure 9). Moreover, T_1_ relaxation in compositionally homogeneous bovine meniscus tissue consisting of bulk fibrocartilage was found to be nonexponential, too, although to a lesser extent than in AC (Figure 10).

To address the observed tendency of T_1_ relaxation in AC toward a single exponential at high fields, we will refer to a particular relaxation model called an exchange‐mediated orientational randomization (EMOR).^11^ Similar to other 3‐pool models,^19^ the EMOR model attributes the water‐^1^H relaxation enhancement in tissue to the proton exchange between bulk water and a pool of protons (termed intermediary protons) consisting of labile macromolecular groups and entrapped water molecules. The efficiency of this mechanism relies on the residence time *τ_ex_* of intermediary protons falling in the microsecond range. In this range, a low‐field water‐^1^H relaxation rate attains its maximum due to a cross‐relaxation coupling between intermediary protons and immobilized protein protons (see Figure 8 in Ref 11). In high fields ($2\pi \nu_{0}\tau_{\mathrm{ex}}>>1$), the efficiency of the EMOR mechanism decreases, and the water‐^1^H relaxation is dominated by a local reorientation of water molecules that has a substantial spectral density at those frequencies. We believe that unlike *τ_ex_*, the local reorientation rate is rather insensitive to the variation of the protein/water composition across morphological zones of cartilage, hence the tendency to a single T_1_ at high fields.

The EMOR model predicts a virtually exponential evolution of the water magnetization after nonselective excitation (the case of FFC). In particular, for the bovine pancreatic trypsin inhibitor as a representative biopolymer system, the deviation from single‐exponential evolution was found to be insignificant at an intermediary proton fraction of 0.01 and hardly detectable at that of 0.1.^11^ Thus, as long as bulk water remains an abundant component, the cross‐relaxation coupling between intermediary protons and immobilized protein protons does not appear to add significantly to these sources of a nonexponential relaxation.

The trypsin digestion of cartilage leads to a broader T_1_ relaxation distribution, as it is securely detected through the parameters *σ_g_* and *β* at all B_0_. On ILT histograms, it manifests as a shift of the long‐T_1_ peak toward larger values, which is assigned to the transitional and the radial zones of cartilage (Figure 5), thus suggesting enhanced water mobility in those zones. Trypsin digestion removes proteoglycans (PGs) while keeping the collagen fibrils mostly intact; hence, the increase in water mobility can be related to the depletion of PGs.^21^ On the one hand, it is known that PG in the form of aggrecans is able to uptake 40% weight of water,^22^ so that a considerable amount of water in natural cartilage can be physically entrapped within the aggrecans, and as such, has a slower dynamics compared with the trypsin‐treated cartilage in which the depleted aggrecans are replaced with protein‐free water. On the other hand, PGs are known to interact with collagen fibrils in such a way that they affect the exchange of water between intrafibrillar and extrafibrillar space.^20^ Neither of these mechanisms can be excluded based on the present results.

To take full advantage of the FFC NMR relaxometry, we compare T_1_ relaxation dispersion profiles between naturally treated and enzyme‐treated cartilage. In the presence of a T_1_ distribution, practitioners of the FFC relaxometry usually analyze dispersions of individual T_1_ components after first decomposing *M*(*t*) on the time scale (such as through a bi‐exponential fit). Our approach is quite opposite: We want to discriminate between components on the frequency scale through decomposition of the average relaxation rate dispersion, $<R_{1}>\left(\nu_{0}\right)$, which calls for estimates of $<R_{1}>$. We analyze such a dispersion as a sum of simple Lorentzian dispersions, which give us access to the correlation time distributions, $g({\tilde{\tau }}_{c})$, according to Equation 4. Although we do not have a dynamic model to verify the obtained $g({\tilde{\tau }}_{c})$, we expect consistent, general direction in which the mean value $<{\tilde{\tau }}_{c}>$ may be changing on enzymatic treatment of cartilage. Such a trend was indeed found for dispersion profiles based on the geometric mean T_1_ (Figure 7), which repeatedly showed an increase in $<{\tilde{\tau }}_{c}>$ after trypsin digestion. However, this contradicts the conclusion that trypsin digestion enhances water mobility in the transitional and the radial zones. Moreover, no trend was found for other measures of average relaxation: the arithmetic mean T_1_ and T_1,_ *_mono_*. It must be concluded, therefore, that this formalism cannot provide reliable information on molecular mobility unless true $<R_{1}>$ values are supplied over the entire frequency range.

## 5 CONCLUSIONS

Relating NMR relaxation behavior to osteoarthritis‐induced cartilage degradation requires a thorough understanding of relevant biochemical features of OA. According to Ref 23, the early‐stage OA can be characterized “as increased water contents, slightly decreased PGs and reorganized collagens.” Seeking a suitable relaxation characteristic that would be sensible to these particular changes in cartilage biochemistry, we investigated T_1_ relaxation in bovine AC at low magnetic fields (B_0_ < 600 mT) using the FFC technique. The early‐stage OA conditions were simulated by enzymatic digestion of cartilage with trypsin.^21^ Using the low fields reveals a T_1_ relaxation‐time distribution that is attributed to morphological zones of articular cartilage—a benefit of the low‐field relaxometry as opposed to the single‐exponential T_1_ relaxation typically observed in stronger fields of 1 T and above.

It was found that the T_1_ distribution width increased significantly after trypsin digestion of cartilage. A general discussion of this result led us to the conclusion that it could indeed be explained by the biochemical changes that are characteristic of the early‐stage OA—particularly by the depletion of PGs. We should recall that this conclusion has the underlying assumption that trypsin treatment reproduces early‐stage OA conditions. Further research on the potential of this parameter (the T_1_ distribution width) to be a biomarker of OA requires validation in trials on articular cartilage in vitro and in vivo; corresponding experiments are currently planned.

The FFC technique is not uniquely suitable for such a type of research; one can envisage T_1_ distribution measurements performed on a low‐field MRI scanner equipped with a permanent magnet. The advantage of the FFC relaxometry over the steady‐field relaxometry is that it introduces through varying B_0_ the second dimension that can be exploited in a complementary way or in its own right to monitor the OA‐induced changes in cartilage.^2^, ^4^ Thus, attempts were made to analyze quasi‐continuous T_1_ dispersion profiles before and after trypsin digestion in terms of elementary Bloembergen‐Purcell‐Pound–type relaxation processes with individual correlation times. The presence in the T_1_ spectrum of the components that are comparable or even shorter than the field‐switching time of the FFC instrument makes the results of such analysis ambiguous; thus, its potential in providing information on molecular mobility in cartilage in terms of *τ_c_* remained limited. However, its feasibility is likely to be demonstrated for most other tissues that possess much longer relaxation times.

The methods that use FFC relaxometry in combination with MRI offer access to spatially resolved T_1_ dispersion of human tissue in vivo. Recently, whole‐body FFC‐MRI scanners have been demonstrated and are now capable of imaging at 0.06 T and 0.2 T, with the relaxation field varying down to 10 kHz.^24^, ^25^ This newly accessible hardware can be exploited noninvasively to gain information on OA‐induced changes in human cartilage, including suitably adapted multicomponent analysis of T_1_ relaxation.

## References

[1] Xia Y , Momot K . editors. Biophysics and Biochemistry of Cartilage by NMR and MRI. Cambridge, United Kingdom: The Royal Society of Chemistry 2017;731.

[2] Broche LM , Ashcroft GP , Lurie DJ . Detection of osteoarthritis in knee and hip joints by fast field‐cycling NMR. Magn Reson Med. 2012;68:358–362. 22161576 10.1002/mrm.23266

[3] Rössler E , Mattea C , Stapf S . NMR dispersion investigations of enzymatically degraded bovine articular cartilage. Magn Reson Med. 2015;73:2005–2014. 24824480 10.1002/mrm.25292

[4] Rössler E , Mattea C , Saarakkala S , et al. Correlations of low‐field NMR and variable‐field NMR parameters with osteoarthritis in human articular cartilage under load. NMR Biomed. 2017;30:e3738. 10.1002/nbm.3738 28543921

[5] Rössler E , Mattea C , Stapf S . Feasibility of high‐resolution one‐dimensional relaxation imaging at low magnetic field using a single‐sided NMR scanner applied to articular cartilage. J Magn Reson. 2015;251:43–51. 25557862 10.1016/j.jmr.2014.10.014

[6] Provencher SW . CONTIN: a general purpose constrained regularization program for inverting noisy linear algebraic and integral equations. Comput Phys Commun. 1982;27:229.

[7] Borgia GC , Brown R , Fantazzini P . Uniform‐penalty inversion of multiexponential decay data. J Magn Reson. 1998;132:65–77. 9615412 10.1006/jmre.1998.1387

[8] Venkataramanan L , Song Y‐Q , Hürlimann MD . Solving Fredholm integrals of the first kind with tensor product structure in 2 and 2.5 dimensions. IEEE Trans Signal Process. 2002;50:1017–1026.

[9] Petrov OV , Stapf S . Parameterization of NMR relaxation curves in terms of logarithmic moments of the relaxation time distribution. J Magn Reson. 2017;279:29–38. 28437715 10.1016/j.jmr.2017.04.009

[10] Rossman LA . Design stream flows based on harmonic means. J Hydraul Eng. 1990;116:946–950.

[11] Halle B . Molecular theory of field‐dependent proton spin‐lattice relaxation in tissue. Magn Reson Med. 2006;56:60–72. 16732594 10.1002/mrm.20919

[12] Hamilton AJS . Uncorrelated modes of the non‐linear power spectrum. Mon Not R Astron Soc. 2000;312:257–284.

[13] Rössler E , Mattea C , Mollova A , Stapf S . Low‐field one‐dimentional and direction‐dependent relaxation imaging of bovine articular cartilage. J Magn Reson. 2011;213:112–118. 21962910 10.1016/j.jmr.2011.09.014

[14] Rössler E . Ortsaufgelöste Niederfeld‐NMR‐Untersuchungen der Wasser‐ und Proteindynamik an Knorpelgewebe sowie dessen Bestandteilen zur Charakterisierung von degenerativen Erkrankungen [Ph.D. Thesis]. Ilmenau University of Technology; 2017.

[15] Wang N , Chopin E , Xia Y . The effects of mechanical loading and gadolinium concentration on the change of T_1_ and quantification of glycosaminoglycans in articular cartilage by microscopic MRI. Phys Med Biol. 2013;58:4535–4547. 23760174 10.1088/0031-9155/58/13/4535 PMC3732659

[16] Reiter DA , Lin PC , Fishbein KW , Spencer RG . Multicomponent T_2_ relaxation analysis in cartilage. Magn Reson Med. 2009;61:803–809. 19189393 10.1002/mrm.21926 PMC2711212

[17] Petrov OV , Rössler E , Mattea C , et al. Low‐field NMR relaxation times distributions and their magnetic field dependence as a possible biomarker in cartilage In: Eskola H, Väisänen O, Viik J, Hyttinen J, editors. IFMBE Proceedings. Basel, Switzerland: Springer Nature; 2018:952–955.

[18] Sunde EP , Halle B . Mechanism of ^1^H–^14^N cross‐relaxation in immoblized proteins. J Magn Reson. 2010;203:257–273. 20163976 10.1016/j.jmr.2010.01.008

[19] Calucci L , Forte C . Proton longitudinal relaxation coupling in dynamically heterogeneous soft systems. Prog Nucl Magn Reson Spectrosc. 2009;55:296–323.

[20] Torzilli PA . Influence of cartilage conformation on its equilibrium water partition. J Orthop Res. 1985;3:473–483. 4067706 10.1002/jor.1100030410

[21] Rojas FP , Batista MA , Lindburg CA , et al. Molecular adhesion between cartilage extracellular matrix macromolecules. Biomacromol. 2014;15:772–780. 10.1021/bm401611b PMC3983133 24491174

[22] Prudnikova K , Yucha RW , Patel P , et al. Biomimetic proteoglycans mimic macromolecular architecture and water uptake of natural proteoglycans. Biomacromol. 2017;18:1713–1723. 10.1021/acs.biomac.7b00032 28398752

[23] Wang K , Wu J , Day R , Kirk TB , Hu X . Characterizing depth‐dependent refractive index of articular cartilage subjected to mechanical wear or enzymic degeneration. J Biomed Optics. 2016;21:095002. 10.1117/1.JBO.21.9.095002 27626900

[24] Ross PJ , Broche LM , Davies GR , Lurie DJ . A fast‐field‐cycling MRI system for clinical applications In Proceedings of the 34th Annual Meeting of ESMRMB; 2017; Barcelona, Spain. Abstract 56.

[25] Lurie DJ , Ross PJ , Broche LM . Techniques and applications of field‐cycling magnetic resonance in medicine In: Kimmich R, ed. Field‐Cycling NMR Relaxometry: Instrumentation, Model Theories and Applications. London, United Kingdom: Royal Society of Chemistry; 2018:563.

